# Derisking translational research in India through the Indian Council of Medical Research (ICMR) network for phase 1 clinical trials and public-private partnerships

**DOI:** 10.1136/bmjopen-2025-111850

**Published:** 2026-07-10

**Authors:** Aruvi Poomali, Jerin Jose Cherian, Nikhil Benroy Noronha, Aparna Mukherjee, Taruna Madan Gupta

**Affiliations:** 1 Development Research, Indian Council of Medical Research, New Delhi, India; 2 Department of Global Public Health, Karolinska Institutet, Stockholm, Sweden

**Keywords:** Clinical trials, Clinical Trial, Randomized Controlled Trial, HEALTH ECONOMICS, Research Design

## Abstract

**Abstract:**

**Importance:**

Phase 1 clinical trials fall at a critical intersection of translational research and drug development. With a high failure rate at this stage, only enterprises capable of substantial risk tolerance and heavy investment can step into translational medicine. Academic and public sector researchers in India often lack expertise in early-phase clinical trials. This, along with a lack of dedicated infrastructure, poses some unique challenges in the early-phase drug development process. In this paper, the authors explore public-private partnership (PPP) models of pharmaceutical innovation and propose the establishment of national clinical trial networks as a solution to derisk the industry by jointly undertaking translational research of innovations addressing public health priorities.

**Observations:**

PPPs offer an alternative approach to overcome the challenges associated with early-phase clinical trials. By fostering collaboration between public and private entities, PPPs leverage the strengths of public institutions, which can provide infrastructure, funding and scientific expertise, while private companies contribute their experience in clinical trial management and commercialisation. This is of particular importance in the development of drugs of national health priority.

**Conclusion and relevance:**

PPPs offer a promising strategy to expedite early-phase clinical trials in India by promoting collaboration, diversification and innovation. By acknowledging and mitigating the inherent risks, public and private stakeholders can work together to develop effective and accessible treatments for patients in need.

## Introduction

 India has come a long way to be recognised today as the pharmacy capital of the world. This has been a consequence of a series of policy reforms. The introduction of the Patent Act of 1970 changed the face of the Indian pharmaceutical industry resulting in an increase in the number of domestic firms that entered generic medicine manufacturing.^1^ Following the economic liberalisation of 1991, the influx of foreign players led to a competitive market which drove excellence. The adoption of the Trade-Related Aspects of Intellectual Property Rights agreement also caused domestic firms to start investing in research and development (R&D).^2^ The R&D expenditure by Indian pharmaceutical companies towards novel products steadily increased until 2005 and has since plateaued at around 5% of revenue.^3^

The New Drugs and Clinical Trials (NDCT) Rules 2019 requires that investigational products intended for clinical trials be manufactured in accordance with Good Manufacturing Practices (GMP).^4^ While India has developed substantial capacity in GMP-compliant manufacturing, preclinical research and late-phase clinical trials, significant gaps remain in translating promising proof-of-concept discoveries into early-phase trials. These gaps are prominent for products addressing national health priorities or unmet medical needs where commercial incentives may be limited.^5^ Strengthening early-phase clinical trial capacity is therefore necessary to ensure that innovations from Indian academia, start-ups and public research institutions can progress. In this article, we examine the translational bottlenecks that impede early clinical development in India and discuss how public-private partnerships (PPPs) and the establishment of clinical trial networks can derisk innovation, strengthen national capacity and accelerate the development of priority health technologies.

## Current Indian ecosystem for translational research

India manufactures about 60 000 different generic medicines across 60 therapeutic categories and accounts for 20% of the global supply of generics.^3 6 7^ With about 3000 pharmaceutical companies and 10 500 manufacturing facilities, the intense competition between generic medicine manufacturers has driven the price of medicines to one of the lowest in the world.^8^ India has also been catching up on PPPs that have yielded quite a few success stories for Indian pharmaceutical companies and biotech start-ups.^3 9^ The credit lies with an ecosystem with capacity ranging from GMP facilities to late-phase clinical development. More recently, this ecosystem has received tremendous support through the establishment of clinical trial networks and partnerships to develop new molecules through various phases of clinical development.^9 10^ In the following paragraphs we describe the capacities of the current Indian ecosystem towards GMP manufacturing, pre-clinical development, early-phase and late-phase clinical development.

### Regulatory reforms

With the NDCT Rules 2019, the Indian regulatory landscape sets the ground rules for pharmaceutical innovation.^4^ The NDCT Rules 2019 fosters the development of innovative products by having accelerated approval processes for products addressing unmet medical needs, empowering ethics committees, and clearly demarcating academic trials from regulatory trials.^11^ Regulations of medical devices, biosimilars, global regulatory harmonisation and registrations of ethics committees and contract research organisations are expected to position Indian biopharma innovations against global players.^12^ The regulatory ecosystem in India has undergone major transformation in the recent past, which has led to a favourable landscape for domestic innovation.

### GMP manufacturing

**Figure 1:**
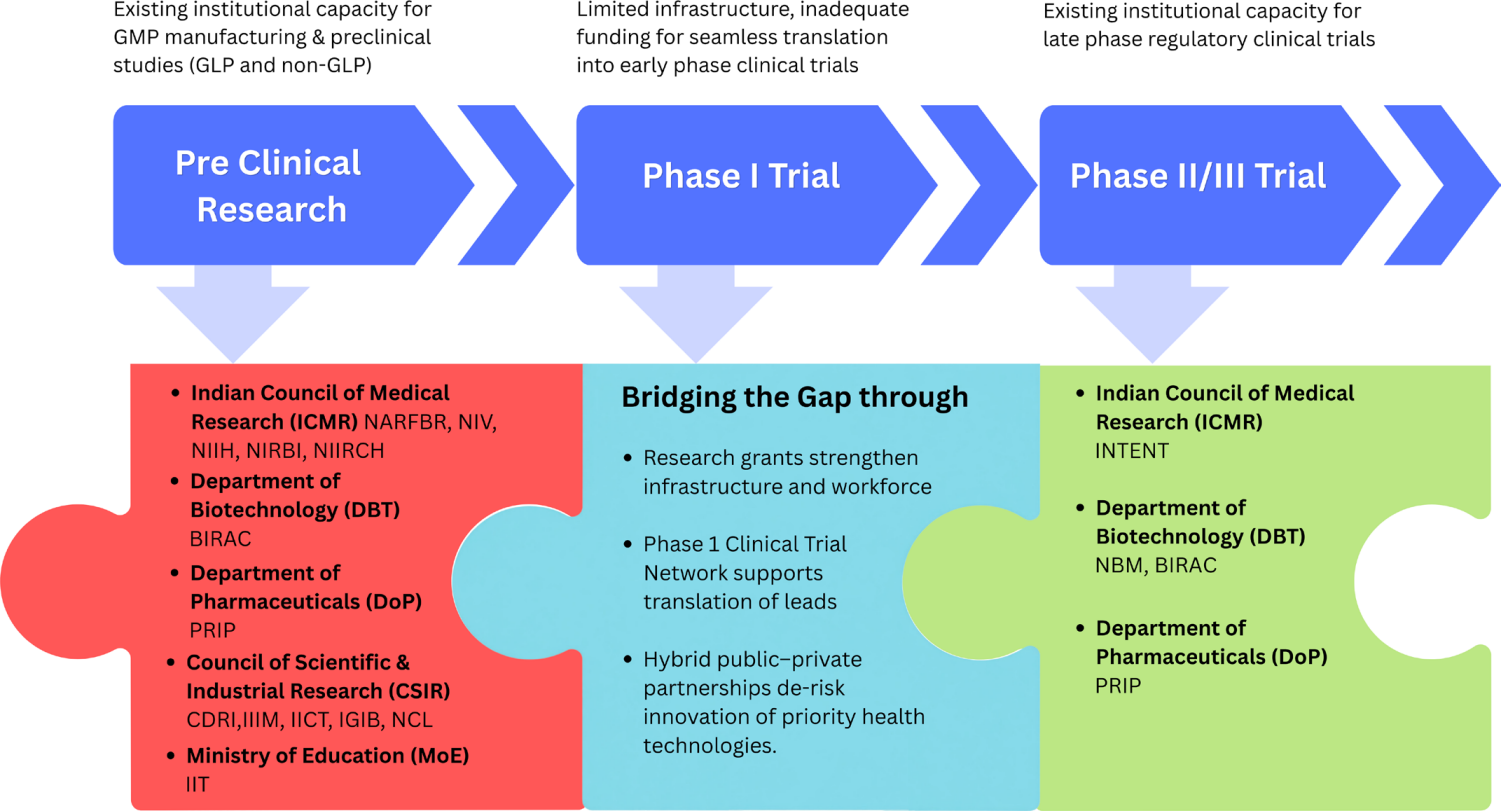
Bridging the gap in translational research: grants, clinical trial networks and public-private partnerships (PPP) derisk and bridge the gap in the innovation of priority health products. BIRAC, Biotechnology Industry Research Assistance Council; CDRI, Central Drug Research Institute; GLP, Good Laboratory Practice; GMP, Good Manufacturing Practice; IGIB, Institute of Genomics and Integrative Biology; IICT, Indian Institute of Chemical Technology; IIIM, Indian Institute of Integrative Medicine; IIT, Indian Institute of Technology; INTENT, Indian clinical Trial Education Network; NARFBR, National Animal Resource Facility for Biomedical Research; NIV, National Institute of Virology; NIIH, National Institute of Immunohaematology; NIRBI, National Institute for Research in Bacterial Infections; NIIRCH, National Institute for Research in Reproductive and Child Health; NCL, National Chemical Laboratory; NBM, National Biopharma Mission; PRIP, Promotion of Research and Innovation in Pharma MedTech sector.

The Indian GMP capacity ranges from its bulk Active Pharmaceutical Ingredient (API) manufacturers and other contract manufacturing organisations in the private sector to specialised public sector research laboratories like the Institute of Chemical Technology, Council of Scientific and Industrial Research–Central Drug Research Institute (CSIR–CDRI), CSIR– Indian Institute of Integrative Medicine (CSIR–IIIM), CSIR–National Chemical Laboratory (CSIR–NCL), CSIR–Indian Institute of Chemical Technology (CSIR–IICT) and CSIR–Institute of Genomics and Integrative Biology (CSIR–IGIB) (figure 1).

Laboratories like CSIR–CDRI have contributed to the growth of the Indian pharmaceutical industry through the development of laboratory-level processes later scaled up to the industry level by the private industry.^7^ There is also evidence for GMP capacity for specialised products in other deep-tech institutions such as the Indian Institute of Science, Bengaluru, and the Indian Institute of Technology, Mumbai.^9^ API manufacturing in India has received increasing assistance through adoption of international regulatory standards and government schemes for Bulk Drug Parks, Production-Linked Incentives and Revamped Pharmaceuticals Technology Upgradation Assistance.^13^,^15^

### Preclinical development

When it comes to preclinical development, full adherence of India to Mutual Acceptance of Data (MAD) in 2011 has resulted in many facilities with the capability to conduct Good Laboratory Practices (GLP) studies in pharmaceuticals as well as in other sectors.^16^ As per the NDCT Rules 2019, toxicity studies should comply with the norms of GLP.^4^ The GLP laboratories in India with drug development expertise extend from private sector contract research organisations (CROs) to the public sector institutions including the Indian Council of Medical Research (ICMR) institutes—National Animal Resource Facility for Biomedical Research (NARFBR), National Institute of Immunohaematology (NIIH), National Institute of Virology (NIV), National Institute for Research in Bacterial Infections (NIRBI), National Institute for Research in Reproductive and Child Health (NIRRCH), and CSIR–CDRI.

### Clinical development

Following irregularities and unethical practices in clinical trials, a slew of regulatory reforms helped strengthen participant safety through improved informed consent requirements, stricter compensation guidelines and increased regulatory oversight. These reforms initially led to a brief decline in the number of clinical trials conducted in India.^17 18^ However, the trend has been changing with clinical trial registries showing growing numbers in recent years.^19^,^21^ Data from the International Clinical Trials Registry Platform (ICTRP) shows that of these trials, most are in later phases of clinical development reflecting the industry’s affinity towards later development of leads.^3 21^ This has been described by Lo and Siah to be a result of priority setting by equity-only enterprises.^22^ Leveraging public sector support to derisk equity-only translational research could be one of the solutions to this challenge. The number of early-phase clinical trials has been much lower in India than in various countries. During the period from 1999 to 2022, the ICTRP website listed 2006 phase 1 clinical trials from India. However, Sabu *et al* reported that the actual numbers were much lower due to the misclassification of phase 1 trials in such registries.^23^ However, the momentum gained by Indian drug development in the discovery and preclinical research thus enters a valley of death at the point of translation.^5^

Late-phase clinical trials in India have received an added boost with capacity in the form of various clinical trial networks such as ICMR–INTENT (**In**dian clinical **T**rial **E**ducation **N**e**t**work) network, National Biopharma Mission–CHOORD (**C**onsortia of **H**ospitals in the areas of **O**ncology, **O**phthalmology, **R**heumatology and **D**iabetology), and those of CSIR-CDRI, in addition to the pharmaceutical industry.

Therefore, the time is optimum to find solutions to increase translational research in India. It is of importance that these solutions also derisk translational research. Derisking in the context as described in this paper thus implies eliminating the financial risks as well as the risk of product failure of early-phase clinical development of leads of national health priority.

## Challenges to early-phase clinical development of pharmaceutical agents

Establishing safety using phase 1 clinical trials is a regulatory requirement, and there are no robust alternatives to phase 1 clinical trials. For new drug substances discovered or developed in India, data including that from phase 1 clinical trials need to be submitted to the regulator. The clinical trial should also be conducted in accordance with the approved clinical trial protocol and as per the requirements of Good Clinical Practices Guidelines and the provisions of the NDCT Rules 2019.^4 24^ A 2017 paper by Differding reports the success rate of phase 1 clinical trials for Indian companies as 54%, which is nearly as good as the global industry average, even though it takes about twice as much time in India.^3^ Even with this being the acceptable success rate, challenges abound when it comes to the initiation of early-phase clinical trials in India. While Kshirsagar *et al* have previously elaborated on the need to facilitate early-phase clinical trials in India, Bano *et al* have described the wide gap that exists between basic and translational research, which makes drug discovery disjointed from the development pipeline. The authors identified challenges in translational research with slow regulatory approvals, slow patent updates, poor community support, an untrained workforce, inadequate funding and increasing competition as major bottlenecks.^5 10^

Phase 1 clinical trials especially require dedicated infrastructure and trained personnel. The complex regulations associated with these trials add to the delay in their initiation.^4 11 25^ Moreover, once the lead is optimised and preclinical studies are completed, the phase 1 clinical trial is often considered a point of no return from a financial risk standpoint. This is because when a lead transitions from preclinical to clinical development, about 40% of the time and investments have been spent in its development. Besides, 90% of leads that enter clinical development are bound to fail. Therefore, only enterprises with heavy investment in deep tech can afford the risk appetite for translational medicine.^26^ Further, the unknown benefit-risk profile of lead molecules and the unpredictable outcome of later phases of clinical trials leave few takers interested in early-phase clinical trials. These circumstances have led to many Indian biopharmaceutical innovators offshoring their early clinical development. Many of the multinational pharmaceutical companies have given up the traditional ‘fully integrated discovery and development’ model owing to its non-profitability and have explored various other innovation strategies including mergers and acquisitions, alliances, purchase of services and in-licensing.^27^ Today, many of these companies work as knowledge aggregators rather than companies with deep technical capabilities in drug discovery and development.^28^

## Possible strategies from the Indian context

### PPPs for derisking translational research

**Figure 2:**
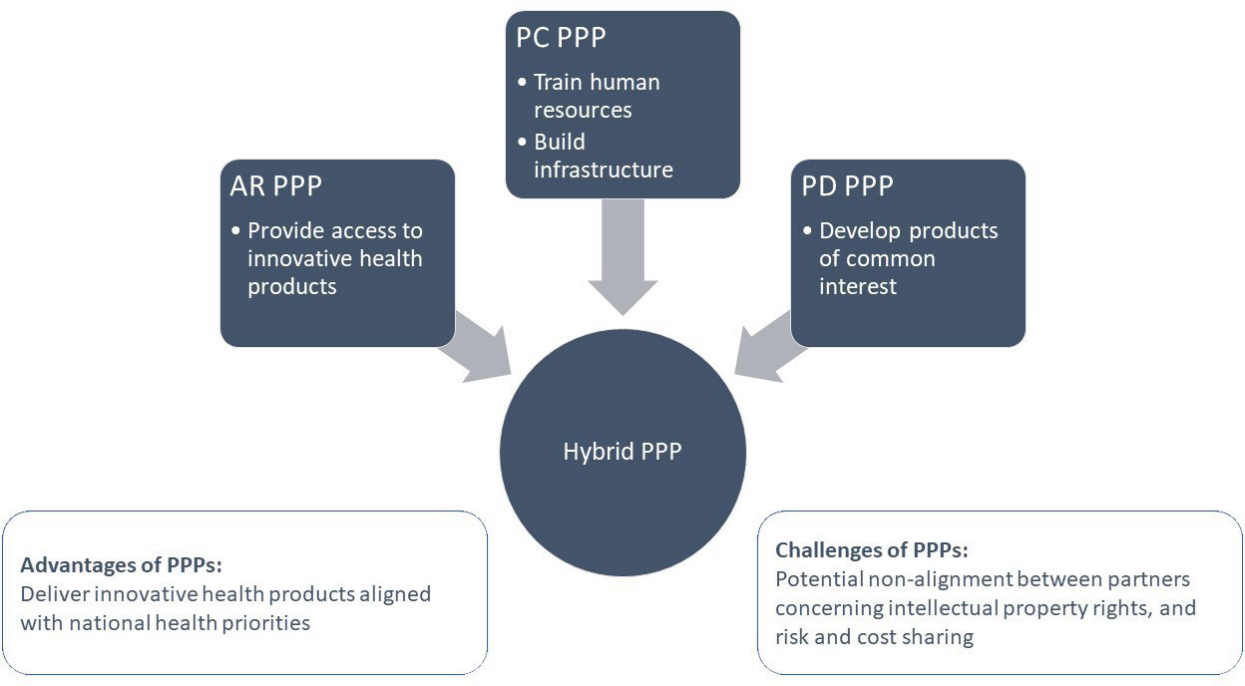
Three models of public-private partnerships (PPPs) in biopharmaceutical innovation. Hybrid public-private partnerships support clinical development of leads and improve access to innovative health products by building infrastructure and training human resources like pre-competitive (PC) PPPs, developing products of common interest like product development (PD) PPPs and improving access to innovative health products like access-related (AR) PPPs.

The clarion call for innovative business models in drug discovery and development has been loud and clear for some time. One of the models that the industry has been willing to explore is open innovation with PPPs.^29 30^ Bagley *et al* define a pharmaceutical PPP agreement as a ‘legally binding contract between a private pharmaceutical enterprise and a public sector agency (usually a research university or a science-based government department) to support research leading to new commercial pharmaceutical and biological products’.^31^ Pharmaceutical PPPs may be broadly categorised into three categories (figure 2).^32^ In pre-competitive (PC) PPPs, the partnership involves building infrastructure and training human resources. Product development (PD) PPPs are those where the partnership is centred around the development of a specific product of common interest. Lastly, access-related (AR) PPPs are usually focused on policy levers which improve access to innovative health products. PC and PD PPPs deal directly with clinical development, while AR PPPs are those concerned with improving access of new medicines to target populations. A combination of these three may be considered hybrid PPPs.

### Clinical trial network for phase 1 studies

The ICMR Network for Phase 1 Clinical Trials, established in 2024, represents a model that incorporates elements of a hybrid PPP framework.^33^ First, the network in its PC PPP efforts has built infrastructure and manpower capacity through core grants to establish Centres for Advanced Research. Manpower trained for medical writing, clinical management of participants, bioanalysis, data management, and regulatory affairs is expected to bolster the capacity in India to take forward high-quality regulatory clinical trials, which would otherwise have been pursued overseas. Through a rigorous selection process, ICMR has identified academic centres across the country with an established track record of conducting early-phase clinical trials. ICMR has augmented the existing infrastructure at these Centres for Advanced Research through a research grant spread over a period of 5 years. Annual workshops that bring together subject experts are being organised to ensure relevant training for the teams. These academic institutions thus play an active role in various stages of translational science from protocol development to implementation of the clinical trials.

Second, ICMR has further established PD PPPs with individual biopharmaceutical innovators. The partners have been identified through applications for co-development. The network comprising four institutions is currently involved in five phase 1 clinical trials in oncology, vaccine, and cell and gene therapy. These trials are of lead molecules that are investigational new drug (IND) ready and are of national health priority. The molecules which have thus entered early-phase clinical development in the network have the opportunity for further regulation-compliant late-phase development through the ICMR INTENT network for the phase 2 and phase 3 clinical trials. 75 sites have been established under this network. Additionally, MedTech Mitra, a strategic initiative launched by the Ministry of Health and Family Welfare, is jointly coordinated by ICMR and the Central Drugs Standard Control Organisation (CDSCO) under the guidance of NITI Aayog (National Institution for Transforming India) to empower MedTech innovators and advance healthcare solutions. More than 360 innovators have used the platform in the last 1 year and many of whom are currently being supported for clinical evaluation at various ICMR–INTENT network sites.^34^ These biopharmaceutical innovators are comprised of public enterprises, start-ups, and nascent pharmaceutical companies.

Lastly, the network has established terms of social responsibility with the innovators, focusing on reduced prices of products following market authorisation, subsidised prices for public sector procurement, etc., like a typical AR PPP. The network thus cultivates a hybrid PPP model that derisks early-phase clinical development of products that are of national health priority through capacity building, funding phase 1 clinical trials, and promoting access to innovative health technology.

## Existing PPP models and trial networks for pharmaceutical innovation

Some examples of early-phase drug development partnerships and networks from high-income countries that are available in published literature are given below:

1. The biomedical public-private landscape in the Netherlands is represented by three institutions—Top Institute Pharma, the Centre for Translational Molecular Medicine and the BioMedical Materials programme which jointly focus on expedited translation of new scientific findings into clinical proof of concept studies.^35^
2. Product development partnerships have led the R&D for numerous neglected diseases and have taken a few new chemical entities to clinical trials.^36^,^38^ Development of a chemotherapeutic agent for onchocerciasis was of utmost priority for the WHO’s Special Programme for Research and Training in Tropical Diseases (TDR) programme. The trials to test the efficacy of the medicine in field settings were funded by Merck, Onchocerciasis Control Programme (OCP) and TDR. TDR’s existing international network helped conduct activities in Africa and South America. TDR further contributed to the protocol and supported applied research on onchocerciasis.^39^
3. The Drugs for Neglected Diseases Initiative (DNDi) has also fostered PPP between pharmaceutical companies, governments, and other agencies for neglected diseases.^40^ The DNDi helps discover, develop, and deliver new treatments for neglected patients around the world. Since 2003, the DNDi has developed 13 treatments for six diseases and has 40 ongoing projects in nine disease areas and clinical trials in 28 countries. In addition to the development of treatments, it has also helped build clinical research capacity in these countries.^41^
4. The Innovative Medicines Initiative (IMI)—a PPP between the European Union, represented by the European Commission, and the European Federation of Pharmaceutical Industries Associations—was launched in 2008 to improve the efficiency and effectiveness of the drug development process. A major achievement by the initiative has been to foster a collaboration between public and private partners by providing a neutral platform where all partners are bound by the same rights and responsibilities. The initiative has resulted in projects like the New Drugs for Bad Bugs (ND4BB) where the partners are addressing antimicrobial resistance R&D. Through the Combatting Bacterial Resistance in Europe (COMBACTE) projects, the initiative has set up a network of hundreds of hospitals and laboratories to conduct pan-European trials. The Ebola vaccine projects (EBOVAC) 1 and 2 projects support the clinical trial testing of the Ebola vaccines through phases 1–3. Their conect4children consortium aims to create a network that will deliver high-quality phase 1–4 clinical trials for molecules in different therapeutic areas across all age groups.^42^ By 2018, 119 projects had been launched and the priority areas in the IMI–2 Strategic Research Agenda had been met.^42^
5. In South Korea, the Seoul National University Hospital Clinical Trials Centre was established in 1997 and the new clinical trial authorisation process was introduced in 2002 to streamline the regulatory process, which led to an increase in the number of multinational late-phase trials conducted in the country. The numbers further increased due to government funding promoting the setting up of regional clinical trial centres. This has also increased the number of early-phase clinical trials since then with more than 40 phase 1 and phase 2 studies each initiated in the year 2018.^43^

Similar initiatives in low-income and middle-income countries are given below:

A review of https://clinicaltrials.gov/ showed that clinical trials conducted outside high-income countries are mostly of late-phase trials.^44^ However, some non-high-income countries have been prioritising early-phase drug development by exploring suitable PPP models. With 60% of the world population residing in Asia and a genetically diverse patient pool, industry-sponsored research has been growing in Asia.^45^

1. In 2012, the Government of Malaysia established a non-profit company—Clinical Research Malaysia (CRM)—to establish an ecosystem of clinical research in the country. With a goal to increase the number of clinical trials, the initiatives included setting up more research centres, training investigators and improving Institutional Review Board and Institutional Ethics Committee timelines.^45 46^ To further improve the number of early-phase clinical trials conducted in the country, in 2016, the CRM initiated the Phase 1 Realisation Project (P1RP) to ensure the country’s readiness for early-phase clinical trials, especially first-in-human trials. The project was intended to develop phase 1 clinical trial guidelines, build capacity of regulatory agencies as well as local experts and establish clinical trial units in hospitals and develop an action plan for any crisis that may arise during the trial. The P1RP initiative was wrapped up in 2021. Since then, multiple feasibility studies on first-in-human trials have been conducted by the CRM and there has been an increase in the conduct of phase 1 clinical trials in Malaysia in recent years.^47^
2. In India, the Biotechnology Industry Research Assistance Council (BIRAC) is a not-for-profit public sector enterprise set up by the Department of Biotechnology, Government of India, in 2012.^48^ A mix of PD PPP and PC PPP, it is expected to provide support to the strategic research and innovation capabilities of emerging biotech enterprises that address nationally relevant products. BIRAC co-funded the phase I/II clinical trial of a chimeric antigen receptor (CAR)-T-cell therapy along with ImmunoACT, an Indian Institute of Technology, Bombay start-up, and Advanced Centre for Treatment, Research and Education in Cancer (ACTREC), Tata Memorial Hospital. The therapy received approval from the CDSCO in October 2023.^49^ BIRAC has also funded the development of the novel antimicrobial nafithromycin.^50^
3. The ‘Promotion of Research and Innovation in Pharma MedTech sector (PRIP)’ scheme of the Department of Pharmaceuticals, India, is another PPP model, especially the B–II component of the scheme, wherein the government is inviting applications from Pharma & MedTech companies to support Lab to Market Transition (Technology Readiness Levels 5 to 9), with a financial support of up to Rs 100 Cr/project or 35% of the project cost, whichever is lesser.^51^

## Challenges of PPP models in drug development and possible solutions

Effective PPPs can lead to a better capacity to deliver innovative health products that meet national health priorities. However, a significant hurdle lies in the potential non-alignment of priorities between public and private sector partners. While public entities often prioritise societal impact and access to affordable medicines, equity-based private companies are driven by commercial considerations. This divergence in goals can sometimes lead to disagreements and affect the execution of projects.^3^

Another challenge is the complex interplay of intellectual property (IP) rights. Leveraging public sector funding to derisk clinical development of products should eventually benefit society. PPPs often involve sharing proprietary knowledge and technologies, raising concerns about ownership, licensing and royalty. Disputes over IP can strain collaborations and delay progress. Furthermore, establishing equitable and mutually beneficial IP arrangements is crucial to incentivise innovation, fostering trust and ensuring fair distribution of the benefits.^52^ At the ICMR Network for Phase 1 Clinical Trials, the background IP rights remain with the innovator who also holds the licence. Additionally, negotiations for any new IP developed and licensing are made on a case-to-case basis. The ‘ICMR-Guidelines for Technology Transfer and Revenue Sharing’ and its amendments guide all decisions made in relation to this subject.^53^ While this manuscript focuses on domestic capacity building, broader geopolitical factors—such as regulatory divergence, technology transfer constraints and supply chain dependencies—may influence international collaboration and should be considered in the future evolution of public-private partnership models.

Risk and cost sharing are fundamental aspects of PPPs. Partners need to agree on how to allocate the financial burden and potential risks associated with drug development. This includes defining roles and responsibilities for clinical trials, regulatory approvals, manufacturing and commercialisation. Disagreements over risk and cost sharing can lead to financial strain, delays and even the breakdown of partnerships.^54^ However, increased costs and delayed drug development are inherent risks of such partnerships.

To address these challenges, effective communication, negotiation, and trust-building are essential. Transparent discussions about goals, expectations and potential risks are crucial for aligning priorities and building a strong foundation for collaboration. Clear and well-defined IP policies and agreements can mitigate disputes and encourage knowledge sharing. Additionally, innovative financing mechanisms, such as milestone payments, royalties and risk-sharing agreements, can help equitably distribute the financial burden and incentivise both public and private partners.^55^ Advanced market commitments or volume guarantee agreements can shift some risk back onto the public sector and make developers feel more confident that they will recoup costs from their investments.

## Conclusion

PPPs offer an alternate approach to overcome the challenges associated with early-phase clinical trials in India. By fostering collaboration between public and private entities, PPPs can leverage the strengths of public institutions, which can provide infrastructure, funding and scientific expertise, while private companies can contribute their experience in clinical trial management and commercialisation. This collaborative approach stands in contrast to the traditional competitive model, where companies race to develop new drugs, often leading to duplication of efforts and wasted resources. PPPs can further enable biopharmaceutical companies to diversify their asset portfolios by derisking drug development of molecules with a strong societal impact but unattractive market potential. In conclusion, PPPs offer a promising strategy to expedite early-phase clinical trials in India by promoting collaboration, diversification, and innovation. By acknowledging and mitigating the inherent risks, public and private stakeholders can work together to develop effective and accessible treatments for patients in need. The ICMR Network for Phase 1 Clinical Trials is anticipated to derisk translational research in India through PPPs.
