# Supplementary material for: Derisking translational research in India through the Indian Council of Medical Research (ICMR) network for phase 1 clinical trials and public-private partnerships
Source: BMJ Open. 2026 Jul 10;16(7):e111850. doi: 10.1136/bmjopen-2025-111850 (PMC13358229; doi:10.1136/bmjopen-2025-111850)
Supplement: Supplementary file 1 [file bmjopen-2025-111850.reviewer_comments.pdf]

PEER REVIEW HISTORY

BMJ Open publishes all reviews undertaken for accepted manuscripts. Reviewers are asked to complete a checklist review form and are provided with free text boxes to elaborate on their assessment. These free text comments are reproduced below.

ARTICLE DETAILS

Title (Provisional)

Derisking Translational Research in India through the Indian Council of Medical Research (ICMR) Network for Phase 1 Clinical Trials and Public Private Partnerships

Authors

Poomali, Aruvi; Cherian, Jerin Jose; Noronha, Nikhil Benroy; Mukherjee , Aparna; Gupta, Taruna Madan

---

VERSION 1 - REVIEW

**Reviewer**	**1**
**Name**	**Giannuzzi, Viviana**
**Affiliation**	**Fondazione per la Ricerca Farmacologica Gianni Benzi ONLUS**
**Date**	**17-Mar-2026**
**COI**	**None**

This communication manuscript covers well a relevant clinical research topic in a specific setting a geographic area.

In my view, some sentences like "Establishing safety using First–In–Human(FIH) studies is a regulatory requirement, and there are

no robust alternatives to Phase 1 clinical trials.", GLP, GMP, should be supported by relevant regulatory sources

Importantly, while GLP are mentioned and GMP are argued, GCP are not mentioned at all.

Specific comments:

- the authors could clarify if the aim to focus on PPP or on both PPP and clinical trial networks. This should be made consistent along the whole document, including the aim at the end of the Introduction: "we propose public–private partnerships (PPPs) and establishment of clinical trial networks".

- laws, eg

Patent Act of 1970

NDCT Rules 2019

"the regulatory reforms of 2013"

or other statements with regulatory implications, e.g. "The

complex regulations associated with first–in–human trials" should be fully referenced and included in the references list.

- ABSTRACT: at the end of the "Importance" subsection, the authors should specify if they explored 'national' PPP (ref "the authors explore public–private partnership (PPP) models")

- sub-section "4. Clinical Development": no reason explaining "The regulatory reforms of 2013 had led to a brief decline in the number of clinical trials conducted

in India" is provided. Such an explanation could be interesting for readers. In addition, no comparison is made with the existing clinical trial networks around the world.

- sub-section "Existing models for innovation": the authors do not specify how they identified the "examples of early phase drug development partnerships and networks from high income countries", as they did below for low and middle income countries.

- among Declarations, the "Ethical Approval and consent to participate" should be revised, because the fact that the manuscript is not an original research article does not justify the ethical approval is required or not.

Reviewer **2**

Name **Stewart, Roy**

Affiliation **Rijksuniversiteit Groningen, Department of Health Sciences, Community and Occupational Medicine, University Medical Center Groningen**

Date **24-Mar-2026**

COI **None**

Manuscript ID: bmjopen- 2025-111850.R1

Manuscript title: Derisking Translational Research in India through the ICMR Network for Phase I Clinical Trials and Public Private Partnerships

Thanks for the opportunity to be part of the review of this manuscript.

In this manuscript, the authors propose public–private partnerships (PPPs) and the establishment of clinical trial networks as a potential solution to enhancing capacity in early-phase clinical development in India.

Although PPPs have been proposed and described for some time(1-3), to the best of my knowledge, no such proposal as the one mentioned in this manuscript has yet been described in relation to clinical trial networks.

What is clear, as far as I understand, is that the ecosystem in India can currently manufacture to Good Manufacturing Practices (GMP) standards, as well as conduct preclinical and late-phase clinical development, and therefore it is proposed by the authors that public–private partnerships (PPPs) and the establishment of clinical trial networks be considered as potential solutions to enhance capacity in early-phase clinical development in India.

Nevertheless, there are a few comments that need to be addressed before this manuscript can be accepted.

1. The authors have demonstrated considerable strengths in their manuscript of the Indian GMP capacity but to an outsider, it is not clear how these organisations and institutions are related to the Indian Council of Medical Research (ICMR). However, the addition of a diagram illustrating the internal structure of the organisations mentioned in the section 'Current Indian ecosystem for translational research' could help to clarify the picture.
2. In identifying the inherent risks, the authors do not adequately address the geopolitical aspects (apart from the aforementioned Trade-Related Aspects of Intellectual Property Rights (TRIPS) Agreement) that may influence cooperation between public and private entities currently operating at the international level, due to measures that may be taken by governments.
3. Please note that the manuscript contains several abbreviations. It would therefore be advisable to include a list of abbreviations at the start of the document.
4. The reference list fails to include the date of the last visit to the web page, for example, reference 4 (this reference displays the message “This site can’t be reached”)

References:

1. Ghanashyam B. Can public-private partnerships improve health in India? Lancet. 2008;372(9642):878–9.
2. Raman V. Do the poor benefit from public-private partnerships in health services - evidence from India. Trop Med Int Health. 2007;12:107–.
3. Delhi V, Mahalingam A. Relating Institutions and Governance Strategies to Project Outcomes: Study on Public-Private Partnerships in Infrastructure Projects in India. J Manage Eng. 2020;36(6).

VERSION 1 - AUTHOR RESPONSE

**Reviewer 1**		Relevant revision made
in my view, some sentences like "Establishing safety using First–In–Human(FIH) studies is a regulatory requirement, and there are no robust alternatives to Phase 1 clinical trials.",	Reference added:<br>NDCT Rules 2019<br>Following sentences inserted in relevant section to support claims with regulatory sources:<br>• “For new drug substances discovered or developed in India, data including that from	New reference: No. 4<br>• Line no. 177-180; page 4<br>• Line no. 137-138; page 4

GLP, GMP, should be supported by relevant regulatory sources	phase 1 clinical trials need to be submitted to the Regulator".<br><br>• "As per the New Drugs and Clinical Trial Rules 2019, toxicity studies should comply with the norms of GLP"<br><br>• "The New Drugs and Clinical Trials Rules 2019 requires the manufacture of new drugs for the purposes of clinical trial to be done in accordance with the provisions of these rules and in accordance with the principles of Good Manufacturing Practices."	• Line no. 81-83; page 2
Importantly, while GLP are mentioned and GMP are argued, GCP are not mentioned at all.	We agree with the comment and have inserted the following sentence to address it: "The clinical trial should also be conducted in accordance with the approved clinical trial protocol and as per requirements of Good Clinical Practices Guidelines and the provisions of the NDCT Rules 2019."	New reference: No. 24<br>Line No. 180-182<br>page 4
- the authors could clarify if the aim to focus on PPP or on both PPP and clinical trial networks. This should be made consistent along the whole document, including the aim at the end of the Introduction: "we propose public–private partnerships (PPPs) and establishment of clinical trial networks".	The authors thank the reviewer for highlighting the need for greater clarity and consistency. We confirm that the manuscript intends to present both public–private partnerships (PPPs) and clinical trial networks as complementary, rather than competing, strategies to strengthen early-phase clinical trial capacity in India.<br><br>• To address this, we have revised the aim in the Introduction to explicitly reflect this dual focus:<br><br>"In this article, we propose both public–private partnerships (PPPs) and the establishment of clinical trial networks as complementary strategies to improve capacity in early-phase clinical development in India."<br><br>• To ensure consistency throughout the manuscript, we have also revised the relevant section heading as follows:<br><br>**"Possible strategies from the Indian context**<br>**a) PPPs for de-risking translational research**<br>**b) Clinical trial networks for Phase I studies"**	• Line 99-90, page 2<br>• Line 211, 213, 230; pages 5,6<br>• Line 270; page 6<br>• Line 232; page 6<br>• Line 41-48; pages 1-2

<table>
<tr><td></td><td>We also changed another heading to “Existing PPP models and trial networks for pharmaceutical innovation”
In addition, we have clarified the relationship between these two approaches in the text, emphasizing that while PPPs and clinical trial networks are conceptually distinct, they may operate synergistically. For instance, “ICMR Network for Phase I Clinical Trials, established in 2024, represents a model that incorporates elements of a hybrid PPP framework.” (Reference 33)
The sentence in the Abstract (Importance section) has been retained, as it introduces the discussion on PPP models for pharmaceutical innovation, which forms an integral component of the manuscript’s broader argument.
These revisions ensure a more consistent framing of PPPs and clinical trial networks as complementary strategies across the manuscript.</td><td></td></tr>
<tr><td>- laws, eg
Patent Act of 1970
NDCT Rules 2019
"the regulatory reforms of 2013"
or other statements with regulatory implications, e.g. "The complex regulations associated with first–in–human trials" should be fully referenced and included in the references list.</td><td>References added for the following:
Patent Act of 1970
NDCT Rules 2019
Regulatory reforms of 2013
The complex regulations associated with first–in–human trials</td><td>Ref: No. 1
Ref: No. 4
Ref: No. 17,18
Ref: No. 4,11, 25</td></tr>
<tr><td>- ABSTRACT: at the end of the "Importance" subsection, the authors should specify if they explored 'national' PPP (ref "the authors explore public–private partnership (PPP) models")</td><td>The authors have clarified in the Abstract section of the manuscript that the clinical trial networks described are of national level. (The national level network is cited in Reference 32)</td><td>Line no. 47; page 2</td></tr>
<tr><td>- sub-section "4. Clinical Development": no reason explaining "The regulatory reforms of 2013 had led to a brief decline in the</td><td>The authors agree that providing context for the observed decline in clinical trial activity following the regulatory reforms would enhance the manuscript. Accordingly, the sentence has been reframed to “Following</td><td>Line 147-150; page 4
Line 270-353; page 6-8</td></tr>
</table>

number of clinical trials conducted in India" is provided. Such an explanation could be interesting for readers. In addition, no comparison is made with the existing clinical trial networks around the world.	irregularities and unethical practices in clinical trials, a slew of regulatory reforms helped strengthen participant safety through improved informed consent requirements, stricter compensation guidelines, and increased regulatory oversight. These reforms initially led to a brief decline in the number of clinical trials conducted in India."<br>• The authors submit that similar existing models of innovation from high income as well as LMICs have been provided in the manuscript.	
- sub-section "Existing models for innovation": the authors do not specify how they identified the "examples of early phase drug development partnerships and networks from high income countries", as they did below for low and middle income countries.	The examples of existing models for innovation for high income countries were all identified by review of published literature. The clarification has been added.	Line 273; page 6
- among Declarations, the "Ethical Approval and consent to participate" should be revised, because the fact that the manuscript is not an original research article does not justify the ethical approval is required or not.	The declaration has been revised to read: "This communications article expresses the authors' opinion on derisking early phase clinical trials with references made to existing published literature. Hence the article does not require ethical approval." This is in alignment with the journal's ethics statement.	Line 707-709; page 18
**Reviewer 2**		
Although PPPs have been proposed and described for some time(1-3), to the best of my knowledge, no such proposal as the one mentioned in this manuscript has yet been described in relation to clinical trial networks.<br>1. Ghanashyam B. Can public-private partnerships improve health in India? Lancet. 2008;372(9642):878–9.<br>2. Raman V. Do the poor benefit from public-private	The authors agree with the reviewer that the PPPs described in the references quoted are primarily in relation to health care services and public infrastructure and currently no clinical trial networks exist in India that cater to early phase clinical trials.	-

partnerships in health services - evidence from India. Trop Med Int Health. 2007;12:107–.<br>3. Delhi V, Mahalingam A. Relating Institutions and Governance Strategies to Project Outcomes: Study on Public-Private Partnerships in Infrastructure Projects in India. J Manage Eng. 2020;36(6).		
1. The authors have demonstrated considerable strengths in their manuscript of the Indian GMP capacity but to an outsider, it is not clear how these organisations and institutions are related to the Indian Council of Medical Research (ICMR). However, the addition of a diagram illustrating the internal structure of the organisations mentioned in the section 'Current Indian ecosystem for translational research' could help to clarify the picture.	We agree that, for international readers, the relationships between the organisations described and the Indian Council of Medical Research (ICMR) may not be obvious. We would like to clarify that, while the institutions discussed in the section belong to the broader national ecosystem, they **are** not structurally or administratively part of ICMR**.** Rather, they operate under different ministries, or as independent entities, with ICMR playing a complementary and facilitative role in advancing translational research.	-
2. In identifying the inherent risks, the authors do not adequately address the geopolitical aspects (apart from the aforementioned Trade-Related Aspects of Intellectual Property Rights (TRIPS) Agreement) that may influence cooperation between public and private entities currently operating at the international level, due to measures that may be taken by governments.	We agree that geopolitical factors—including regulatory divergence, technology transfer restrictions, supply chain dependencies, and evolving national policies—can significantly influence international cooperation between public and private entities.<br><br>We have included the following sentence to highlight this potential risk in the Challenges section: “While this manuscript focuses on domestic capacity building, broader geopolitical factors- such as regulatory divergence, technology transfer constraints, and supply chain dependencies—may influence international collaboration and should be considered in the future evolution of public–private partnership models.”	Lines 375-378; page 9

3. Please note that the manuscript contains several abbreviations. It would therefore be advisable to include a list of abbreviations at the start of the document.	All abbreviations have now been expanded at the instance of first use.	-
4. The reference list fails to include the date of the last visit to the web page, for example, reference 4 (this reference displays the message "This site can't be reached")	The reference list has now been revised to incorporate the date of last visit. The erstwhile reference no. 4, currently no. 6 is currently available for view by the authors. It is the website of the Press Information Bureau by the Government of India. The access may be unfortunately restricted for the reviewer for reasons that is not available to the authors.	-

---

VERSION 2 - REVIEW

Reviewer **1**

Name **Giannuzzi, Viviana**

Affiliation **Fondazione per la Ricerca Farmacologica Gianni Benzi ONLUS**

Date **05-May-2026**

COI

---

references should be checked. the aim of the manuscript could be better explained

---

Reviewer **2**

Name **Stewart, Roy**

Affiliation **Rijksuniversiteit Groningen, Department of Health Sciences, Community and Occupational Medicine, University Medical Center Groningen**

Date **10-May-2026**

COI

I would like to express my thanks and appreciation for the way in which the authors have answered my questions, but I have two comments and one question:

1. The authors have not attempted to produce a diagram illustrating the relationships between the organisations described and the Indian Council of Medical Research (ICMR).

However, I believe that the internal structure of the organisations mentioned in this manuscript could provide the novice reader with a clearer picture; as the authors themselves point out, this article will be of interest to policymakers and academics, as well as business leaders and other stakeholders, particularly those from low- and middle-income countries.

2. The authors state that all abbreviations are now written out in full upon first use, but this does not apply to the abbreviation 'ICMR'. This abbreviation appears in the title, and it takes a while before it is written out in full. The abbreviation 'ICMR HQ' is also not written out in full.

Question:

In light of the responses, the question is whether the authors have adequately discussed the limitations of the manuscript? If there are any limitations to report, the authors should state these clearly in the manuscript.

VERSION 2 - AUTHOR RESPONSE

**Reviewer 1**	
references should be checked.	They have been rechecked, including all hyperlinks.
the aim of the manuscript could be better explained	Suitably revised the last paragraph of the 'Introduction' section to better explain the aim of manuscript.
**Reviewer 2**	
1. The authors have not attempted to produce a diagram illustrating the relationships between the organizations described and the Indian Council of Medical Research (ICMR). However, I believe that the internal structure of the organizations mentioned in this manuscript could provide the novice reader with a clearer picture; as the authors themselves point out, this article will be of interest to policymakers and academics, as well as business leaders and other stakeholders, particularly those from low- and middle-income countries.	A new diagram to illustrate the relationships between the organizations has been inserted.
2. The authors state that all abbreviations are now written out in full upon first use, but this does not apply to the abbreviation 'ICMR'. This abbreviation appears in the title, and it takes a while	All abbreviations and acronyms have been defined at first mention as suggested.

before it is written out in full. The abbreviation 'ICMR HQ' is also not written out in full.	
Question:<br>In light of the responses, the question is whether the authors have adequately discussed the limitations of the manuscript? If there are any limitations to report, the authors should state these clearly in the manuscript.	The authors request the reviewer to consider that the format for Communications does not demand a section on limitations. Hence the section has not been included.
